# *Lactococcus lactis*, an Alternative System for Functional Expression of Peripheral and Intrinsic *Arabidopsis* Membrane Proteins

**DOI:** 10.1371/journal.pone.0008746

**Published:** 2010-01-20

**Authors:** Annie Frelet-Barrand, Sylvain Boutigny, Lucas Moyet, Aurélien Deniaud, Daphné Seigneurin-Berny, Daniel Salvi, Florent Bernaudat, Pierre Richaud, Eva Pebay-Peyroula, Jacques Joyard, Norbert Rolland

**Affiliations:** 1 CNRS, Laboratoire de Physiologie Cellulaire Végétale, UMR5168, Grenoble, France; 2 CEA, DSV, iRTSV, LPCV, Grenoble, France; 3 INRA, Laboratoire de Physiologie Cellulaire Végétale, UMR1200, Grenoble, France; 4 Université Joseph Fourier, Laboratoire de Physiologie Cellulaire Végétale, Grenoble, France; 5 CEA, IBS Institut de Biologie Structurale Jean-Pierre Ebel, Grenoble, France; 6 CNRS, IBS Institut de Biologie Structurale Jean-Pierre Ebel, Grenoble, France; 7 Université Joseph Fourier, IBS Institut de Biologie Structurale Jean-Pierre Ebel, Grenoble, France; 8 CEA, DSV, iBEB, Laboratoire des Echanges Membranaires et Signalisation, St Paul les Durance, France; 9 CNRS, UMR 6191, St Paul les Durance, France; 10 Université Aix-Marseille, St Paul les Durance, France; Instituto Butantan, Brazil

## Abstract

**Background:**

Despite their functional and biotechnological importance, the study of membrane proteins remains difficult due to their hydrophobicity and their low natural abundance in cells. Furthermore, into established heterologous systems, these proteins are frequently only produced at very low levels, toxic and mis- or unfolded. *Lactococcus lactis*, a Gram-positive lactic bacterium, has been traditionally used in food fermentations. This expression system is also widely used in biotechnology for large-scale production of heterologous proteins. Various expression vectors, based either on constitutive or inducible promoters, are available for this system. While previously used to produce bacterial and eukaryotic membrane proteins, the ability of this system to produce plant membrane proteins was until now not tested.

**Methodology/Principal Findings:**

The aim of this work was to test the expression, in *Lactococcus lactis*, of either peripheral or intrinsic *Arabidopsis* membrane proteins that could not be produced, or in too low amount, using more classical heterologous expression systems. In an effort to easily transfer genes from Gateway-based *Arabidopsis* cDNA libraries to the *L. lactis* expression vector pNZ8148, we first established a cloning strategy compatible with Gateway entry vectors. Interestingly, the six tested *Arabidopsis* membrane proteins could be produced, in *Lactococcus lactis*, at levels compatible with further biochemical analyses. We then successfully developed solubilization and purification processes for three of these proteins. Finally, we questioned the functionality of a peripheral and an intrinsic membrane protein, and demonstrated that both proteins were active when produced in this system.

**Conclusions/Significance:**

Altogether, these data suggest that *Lactococcus lactis* might be an attractive system for the efficient and functional production of difficult plant membrane proteins.

## Introduction

Membrane proteins carry out a wide range of functions in vital processes such as cell growth and division, maintaining cell integrity, energy transduction, signal sensing and transduction, cell-cell interactions and transmembrane transport mechanisms. Therefore, they are the most important group of proteins in terms of drug targets (for a recent review, see [1]). Approximately 25% of genes identified in all genomes are known to code for membrane proteins [2] but the vast majority has no assigned function. Several reasons can account for such a situation but it is mainly owing to the experimental difficulties encountered with studies targeted to transmembrane proteins. Despite their functional and biotechnological importance, the study of membrane proteins remains difficult due to their low natural abundance in cells and to their hydrophobicity. Moreover, these proteins are frequently *i*) toxic when expressed into established heterologous systems (bacteria, yeast, insect cells, mammalian cells, xenopus oocytes) *ii*) expressed at a very low level in space-limited membranous environment and *iii*) often mis- or unfolded when expressed. Consequently, although the generation of 3-D structures of soluble proteins has entered a high-throughput stage, less than two-hundred high-resolution 3D structures were obtained so far for transmembrane proteins (http://blanco.biomol.uci.edu/Membrane_Proteins_xtal.html). Moreover, most structures of transmembrane proteins have been solved for proteins from bacteria and archae, and only a few have been solved from eukaryotic systems [1]. Before crystallization, expression of sufficiently high amounts of proteins is the first severe bottleneck in structural studies of membrane proteins.

Food grade lactic acid bacteria (LAB) are increasingly used as oral delivery vehicles for therapeutic and prophylactic proteins (for recent reviews, see [3]–[6]). Among them, *Lactococcus lactis* is nowadays widely used in biotechnology for large-scale production of heterologous proteins [3]–[8]. During the past two decades, remarkable progress has been made toward the development of genetic engineering tools and the molecular characterization of *L. lactis* [9]–[11]. This toolbox now includes transformation, gene integration, gene knockout, conjugation, as well as constitutive and regulated gene expression systems (for review, see [12]). The Nisin Inducible Controlled gene Expression (NICE) system uses the quorum sensing regulatory circuit generated by the antimicrobial peptide nisin and therefore, is suitable for the production of recombinant proteins allowing a fine control of gene expression.

Several modifications of this system have increased its use. When a gene of interest is subsequently positioned under the control of the nisin-inducible promoter P_nisA_ in a plasmid such as pNZ8148, the addition of sub-inhibitory amounts of nisin (0.1–5 ng/ml) to the culture medium induces the expression and the production of the corresponding protein (for review, see [13]).

Various eukaryotic membrane proteins have already been produced using the NICE system in *L. lactis* (for reviews, see [4]–[6], [14], [15]). However, to date, there is no report regarding the ability of this expression system to produce functional plant membrane proteins. The aim of this work was to test the expression, in *L. lactis*, of several peripheral or intrinsic *Arabidopsis* membrane proteins that, in our hands, could not be produced, or in insufficient amount, using more classical heterologous expression systems.

As a model for a peripheral membrane protein, we used the *Arabidopsis* ceQORH protein (chloroplast envelope Quinone OxidoReductase Homologue). This protein was the first protein demonstrated to be addressed to the inner membrane of the chloroplast envelope while lacking a classical and cleavable transit sequence [16]. This protein was also demonstrated to use an alternative targeting pathway for its import into the chloroplast [17]. This peripheral protein is associated to the inner membrane of the chloroplast envelope through electrostatic interactions. However, when expressed in *Escherichia coli*, the protein is mostly recovered in inclusion bodies, thus strongly limiting further functional studies. In addition to this peripheral protein, we also decided to test the expression of five intrinsic *Arabidopsis* membrane proteins. First, we chose to express four of the eight members of the *Arabidopsis* P_1B_-type ATPase subfamily implicated in metal transport, AtHMA1, AtHMA3, AtHMA4 and AtHMA6 (also named PAA1) (for reviews, see [18]–[20]). These P_1B_-type ATPases are highly hydrophobic membrane proteins, which contain six to height predicted transmembrane domains. AtHMA1 and AtHMA6 are associated with the chloroplast envelope and are involved in copper (Cu) transport/delivery into the chloroplast [21], [22]. AtHMA3 and AtHMA4, respectively localized in the tonoplast and in the plasma membrane, are predicted to be involved in zinc (Zn), cadmium (Cd), and/or lead (Pb) transport [23]-[25]. Previous attempts for expressing these ATPases in different expression systems (*E. coli*, insect cells and yeast) were either not successful or disappointing because of the very low production level obtained. The fifth chosen intrinsic membrane protein is the ATP/ADP translocator, AtAATP1, which is localized within the inner membrane of the chloroplast envelope in plants [26] and imports ATP in the chloroplast in exchange of ADP and Pi where Pi is a co-substrate of ADP [27]. AtAATP1 is also a highly hydrophobic membrane protein as it contains 11 or 12 predicted transmembrane helices [28]. The physiological role of this transporter is the energy supply of storage plastids required for starch synthesis, and to allow nocturnal anabolic reactions in chloroplasts [29].

During this work, both peripheral and intrinsic *Arabidopsis* membrane proteins could be produced, solubilized from the crude *L. lactis* membranes and purified at levels compatible with further functional and structural studies. Functional studies on purified ceQORH protein and on recombinant bacteria expressing AtAATP1 revealed that these two proteins were active when expressed in *L. lactis*. Altogether, these data suggest that *L. lactis* is an attractive system for the efficient and functional production of difficult plant membrane proteins.

## Results

### Rationale for the Development of a Cloning Strategy Compatible with Gateway Entry Vectors and *Lactococcus* Expression Vector

The aim of this study was to evaluate the efficiency of the *L. lactis* expression system on several independent plant membrane proteins among which one peripheral and five intrinsic membrane proteins. For further detection and purification of the recombinant proteins, they were tagged with a *Strep*-tag II that was already successfully used for expression of recombinant proteins in Gram-positive bacteria [30] and for the expression and purification of the recombinant plant ATPase AtHMA2 produced in yeast [31]. *Strep*-tag II appears to be an acceptable compromise for efficient affinity purification with good yields of prokaryotic membrane proteins in various systems [15], [32], [33]. It possesses only eight amino acids (Trp-Ser-His-Pro-Gln-Phe-Glu-Lys) with no particular overall charge and is therefore biologically inert. It is proteolytically stable and does not interfere with membrane translocation or protein folding. The presence of *Strep*-tag II at the *C*-terminal extremity of AtHMA2 did not alter the activity of this protein [31]. *Strep*-tag II has a strong binding affinity for an engineered streptavidin derivative called *Strep-*Tactin, which enables fast and simple one-step purification [34]. Finally, this affinity tag is suitable for large-scale or high-throughput applications and different proteins have already been successfully crystallized with *Strep*-tag II.

**Figure 1:**
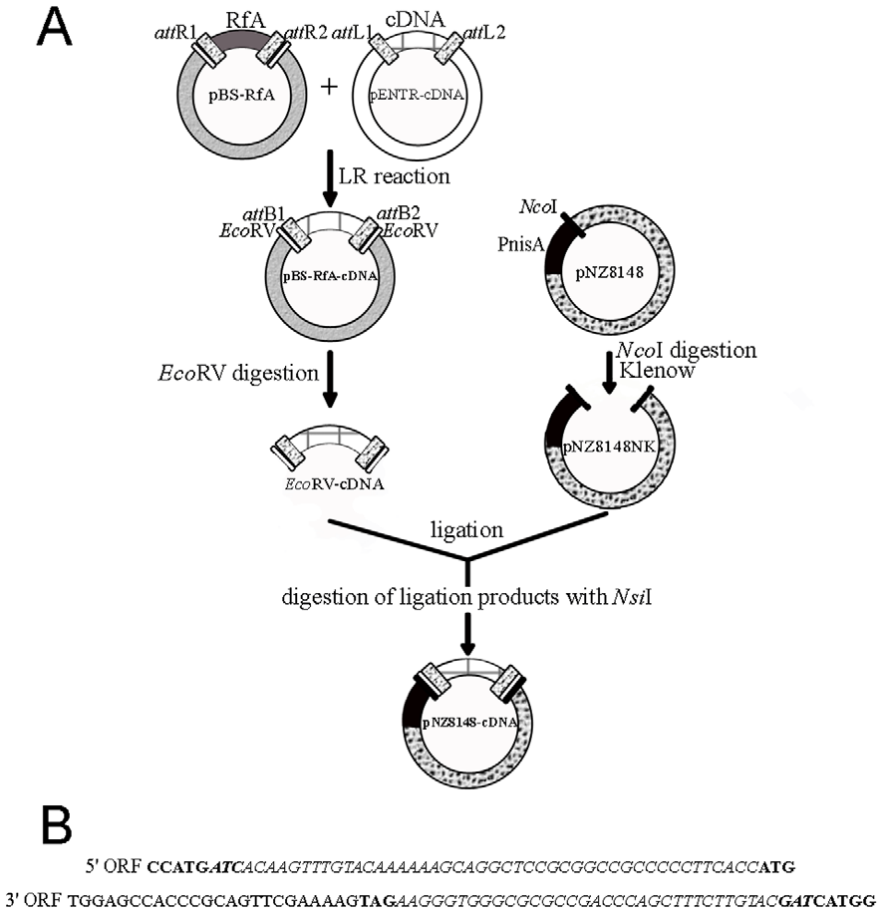
Strategy used to transfer cDNA from Gateway entry vectors to *L. lactis* vectors. A. Overview of the cloning procedure. In this strategy, recombination occurs between the *att*L sites from Gateway entry vectors (containing the cDNA coding for the proteins of interest) and the *att*R sites from a “destination” vector (att: attachment sites). This destination vector (pBS-RfA) is a derivative of pBlueScript which contains the reading frame cassette A (RfA cassette) surrounded by two *Eco*RV sites. By LR recombination, the first step thus generates a “shuttle” vector in which the gene of interest is surrounded by the two *att*B sites and two flanking *Eco*RV restriction sites. In order to generate blunt ends, the nisin inducible vector pNZ8148 is digested by *Nco*I and treated with the Klenow enzyme (pNZ8148NK). The cDNA excised from the “shuttle” vector pBS-RfA-cDNA with *Eco*RV (generating blunt ends) is ligated into the vector pNZ8148NK and placed under the control of the P_nisA_ promoter. Positive selection of recombinant vectors is obtained using digestion of the ligation products with NsiI. **B.** Resulting *N*- and *C*- termini of the ORF. Nucleotide sequences at each side of the ORF (upper panel: 5′ORF and lower panel: 3′ORF). In bold, the ATG (start codon) and TAG (stop codon) of the cDNA. CCATG in bold corresponds to the *Nco*I site after digestion and treatment with the Klenow enzyme; the ATG within the *Nco*I site is in the reading frame of the ATG of the cDNA. The *att*B sites are in italics and the two half-sites resulting from *Eco*RV digestion are in bold italics.

Nowadays, the Gateway technology is widely used to simplify cloning of cDNA into many different expression systems from bacteria to eukaryotic systems. Several libraries are currently available with clones coding for *Arabidopsis* proteins (Orfeome project, [35], [36]) in Gateway compatible vectors. However, the pNZ8148 vector cannot be converted into a Gateway destination vector because of the lack of *Lactococcus* strains able to propagate Gateway vectors. On the other hand, the pNZ8148 cannot be propagated into *E. coli* strains because of problems of DNA instability [37], [38] and the non-complete shut off of the nisin promoter. In an effort to easily transfer cDNAs from Gateway-based *Arabidopsis* cDNA libraries to the *L. lactis* expression vector pNZ8148, we thus established a strategy for rapid transfer of cDNA from Gateway entry vectors into *Lactococcus* nisin-inducible vectors (see details of the strategy in Figure 1A). This process first relies on the previous availability (or the insertion) of cDNAs coding for the proteins of interest into Gateway entry vectors (pENTR-cDNA). A recombination (LR) reaction is then performed which allows to transfer the cDNA from this entry vector to the destination vector (pBS-RfA). The cDNAs are then excised from the pBS-RfA-cDNA vector by digestion with *Eco*RV and ligated into pNZ8148NK. A positive selection of recombinant pNZ8148 vectors can be obtained using digestion of the ligation products with *Nsi*I, a restriction site that is formed by religation of the empty vector (Figure 1A).

It is important to note that using such a cloning strategy maintains the correct reading frame within the fusion-protein sequence since the ATG present in the *Nco*I site of the original pNZ8148 vector is systematically in frame with the initiation codon of the cloned cDNA (Figure 1B). More importantly, the Gateway recombination sequences being translated in *N*-terminus of the recombinant proteins, all proteins are translated from the same 17 first codons and thus share the same *N*-terminal sequence (Figure 1B). In other words, this allows to totally abolish known impact of the diversity of the very first codons on the production level and stability of the produced recombinant proteins [14], [39]. The presence of *Eco*RV restriction sites in AtHMA3, AtHMA4 and AtHMA6 cDNA sequences could easily be circumvented by the use of partial restriction of donor plasmids. However, and as discussed below, several cDNAs were also cloned using a more classical cloning strategy (see materials and methods) to test for the impact of this additional *N*-terminal sequence (resulting from the use of the Gateway entry vectors, see Figure 1B) on the stability or production level of the produced proteins.

### Expression in *Lactococcus lactis* of Plant Peripheral and Intrinsic Membrane Proteins

**Figure 2:**
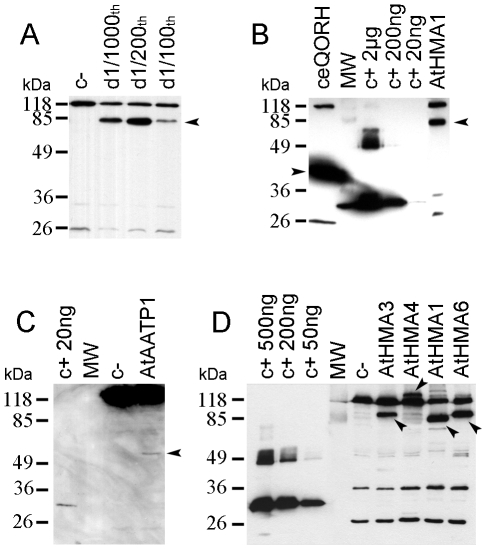
Expression of plant membrane proteins in *L. lactis*. A. Impact of nisin concentration on the expression level of AtHMA1. Production of the AtHMA1 protein after induction by 1/1000^th^, 1/200^th^ and 1/100^th^ dilution of nisin in culture medium. B. Production of the ceQORH and AtHMA1 proteins. C. Production of the AtAATP1 protein. D. Production of the four Arabidopsis P_1B_-ATPases. Total membrane proteins (15 µg for panels A and D, 10 µg for panel B and 50 µg for panel C), were separated in a 10% SDS-PAGE and analyzed by western blot performed using an HRP conjugate specific to the *Strep*-tag II. Arrows indicate the positions of the expressed proteins. In panels B and C, expressed proteins contain an additional *N*-terminal sequence resulting from the translation of the *att*B sites. In panels A and D, c- means crude membrane proteins derived from bacteria containing the empty pNZ8148 vector. In panels B, C and D, defined amounts of a positive control protein (c+, *Strep*-tag II protein) were loaded to estimate the expression levels of the recombinant proteins.

After cloning all the cDNAs into the nisin-inducible vector pNZ8148, we tested the expression, in *L. lactis*, of the peripheral ceQORH protein, and of the five intrinsic membrane proteins: the ATP/ADP translocator (AtAATP1) and 4 P_1B_-type ATPase family (AtHMA1, AtHMA3, AtHMA4, AtHMA6). Bacteria containing the empty pNZ8148 vector were systematically used as negative control (Figure 2A, 2C and 2D, lanes “c-”) to validate the nature of the detected signals.

For every strain, we first determined the optimal concentration of nisin required to induce expression of the corresponding recombinant protein. As a representative experiment, the impact of various dilutions of the nisin solution is shown for cells expressing AtHMA1 (Figure 2A). As can be deduced from this experiment, a dilution of 1/200^th^ was found to be optimum for the production of the HMA1 protein. It is important to note that, since this induction is carried out with the supernatant of *L. lactis* strain producing nisin, the optimal concentration of nisin used for induction was to be determined for every round of nisin production. This problem can be avoided with the use of commercially available nisin stocks. The time of induction was also tested and the protein production level was found to be two to three-times higher (data not shown) using 4 h at 30°C when compared to an overnight induction at 20°C.

For the peripheral ceQORH protein, the disruption of *Lactococcus* cells, performed using the French Press, allowed to extract 5 to 10 mg of crude membrane proteins per liter of culture medium. The ceQORH protein was recovered in the membrane protein fractions where it stands for 20 to 30% of the total membrane proteins (Figure 2B, see also Figure S1).

For the five intrinsic proteins, due to the lower amount of produced recombinant proteins, the yield of crude membrane proteins was to be enhanced. The cell breakage was thus performed using a cell disruptor, which led to an extraction of around 3 to 6 times more (30 mg) crude membrane proteins per liter of grown bacteria. This difference in yield derives from the higher pressure delivered by the cell disruptor when compared to that obtained using the French Press, and thus from an enhanced level of cell breakage.

These all five proteins were successfully expressed in *Lactococcus* and were all recovered in the membrane protein fraction. The levels of production ranged from 0.2% of total membrane proteins for AtAATP1 (Figure 2C) to 1–3% of total membrane proteins for AtHMA1, AtHMA3 and AtHMA6 (Figure 2B and 2D). The AtHMA4 protein was produced in lower amount when compared to the three others ATPases and accumulates to a maximum of 0.2% of total membrane proteins (Figure 2D).

**Figure 3:**
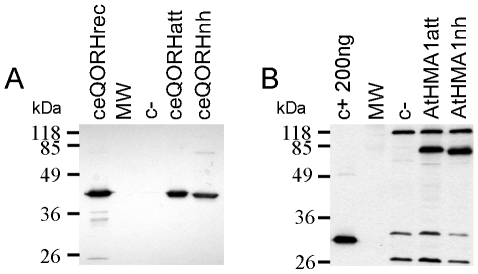
Impact of the *att*B sites on the production level of membrane proteins in *L. lactis*. A. Production of the ceQORH protein using the Gateway compatible (ceQORHatt) or the classical (ceQORHnh) cloning strategies. Control protein: 1 µg of ceQORH rec corresponding to the recombinant ceQORH protein produced in *E. coli* (Miras et al., 2002). B. AtHMA1 production using the Gateway compatible (AtHMA1att) or the classical (AtHMA1nh) cloning strategies. Control protein: 200 ng of the 28 kDa *Strep*-tag II protein. Total membrane proteins (5 µg for panel A and 15 µg for panel B) were separated in a 10% SDS-PAGE and analyzed by western blot using a polyclonal antibody raised against the ceQORH protein (panel A) or an HRP conjugate specific to the *Strep*-tag II (panel B). c-, crude membrane proteins derived from bacteria containing the empty pNZ8148 vector.

As described above, the cloning strategy based on Gateway entry vectors induced the addition of *att*B sites (48 nucleotides) in-between the original vector ATG (within the *Nco*I site) and the initiation codon of the cDNA (Figure 1B). In order to check whether the insertion of this additional sequence affects the expression level of the proteins, two cDNAs (ceQORH and AtHMA1) were also cloned using a more classical strategy [14]. Similar expression experiments were then performed with strains harboring these two constructs (Figure 3). The observed difference in the size of ceQORH (Figure 3A) and AtHMA1 (Figure 3B) confirmed the presence/absence of the *att*B sites (upper and lower bands, respectively). Interestingly, both ceQORH and AtHMA1 were produced at similar levels in the presence or in the absence of the *N*-terminal extension resulting from the translation of the *att*B sites (Figures 3A and B). This result demonstrates that the presence of the *att*B sites in the vector sequence does not affect the production level of these two proteins.

### Solubilization and Purification of the Peripheral ceQORH Protein

**Figure 4:**
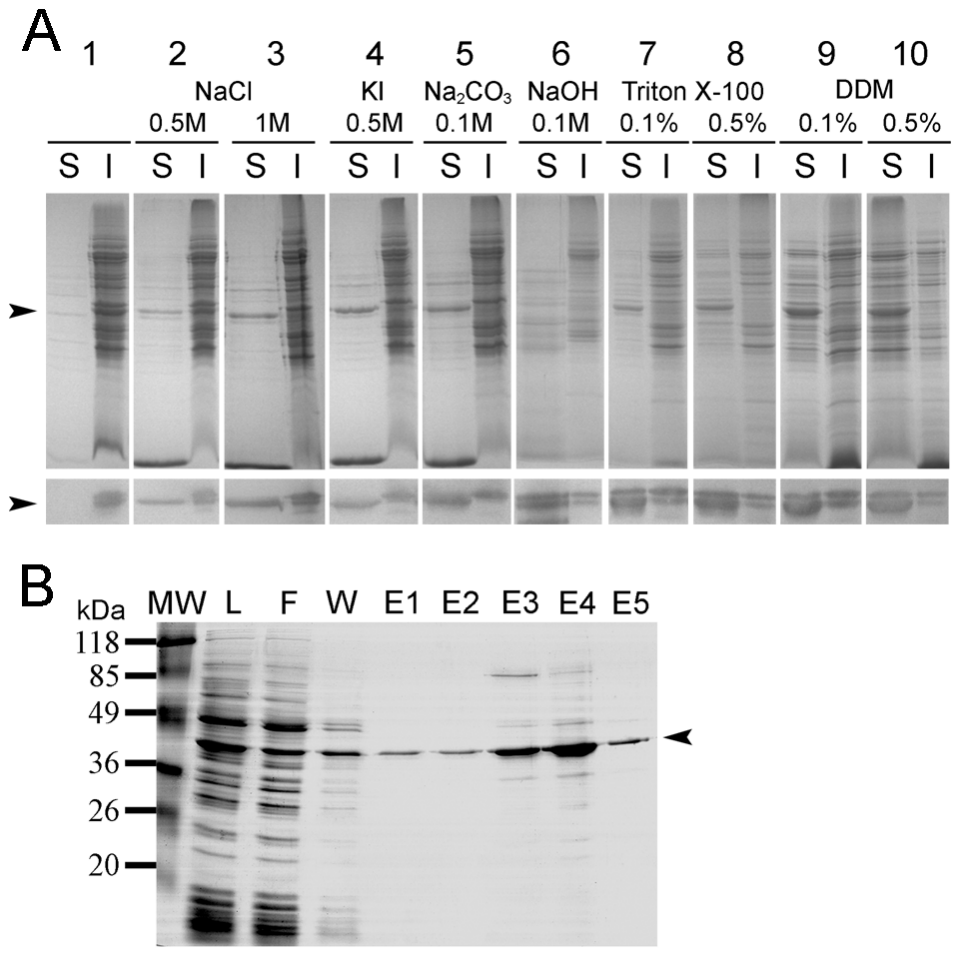
Solubilization and purification of the recombinant ceQORH protein produced in *L. lactis*. A. Impact of salts, pH and detergents on the solubilization of the ceQORH protein from crude *Lactococcus* membrane proteins. Treatments of membrane proteins (1 µg/µL) with various concentrations of salt, detergent or NaOH are described in the Materials & methods section. Solubilized proteins (S) were separated from insoluble membrane proteins (I) by centrifugation. Proteins were analyzed by Coomassie blue-stained SDS-PAGE (upper panel) and by western blot (lower panel) performed using the anti-ceQORH antibody (lanes 1, 2, 4) or a HRP conjugate specific to the *Strep*-tag II (lanes 3, 5–10). B. Purification of the recombinant ceQORH protein on a *Strep*-Tactin Sepharose matrix. Membrane proteins solubilized with 1 M NaCl and desalted on a PD10 column (L) were loaded on the column containing the affinity matrix; F, flowthrough; W, washing fraction; E1 to E5, elution fractions. Aliquots (20 µL) of all fractions were loaded on a 12% SDS-PAGE further stained with Coomassie Blue. The arrow indicates the ceQORH protein.

As mentioned above, the natural ceQORH protein is a chloroplast protein that interacts with the inner envelope membrane through electrostatic interactions [16]. In this context, it is important to note that the *Arabidopsis* ceQORH protein, when produced in *E. coli*, was mostly recovered as inclusion bodies and never found to be associated with the bacterial membrane [16]. On the contrary, the recombinant ceQORH protein produced in *Lactococcus* was recovered in the membrane fraction. Different experiments were thus performed on the purified *Lactococcus* membranes containing the recombinant ceQORH protein to characterize the nature of this interaction with the membrane. Alkaline extractions with Na_2_CO_3_ (Figure 4A, lane 5) or NaOH (Figure 4A, lane 6) induced a release in the soluble phase of the major part of the ceQORH protein. As for the natural protein, salt treatments with NaCl (0.5 and 1 M) or KI (0.5 M) also induced a partial release of the ceQORH protein in the soluble phase (Figure 4A, lanes 2, 3, 4). Treatments with detergents were also performed using 0.1% and 0.5% (w/v) Triton X-100 or DDM (n-dodecyl-β-maltoside) which also induced a partial solubilization of the ceQORH protein (Figure 4A, lanes 7, 8, 9, 10). Taken together, these results indicate that the ceQORH protein interacts with the *Lactococcus* membrane through electrostatic interactions and thus behaves as the natural chloroplast envelope protein [16].

In order to determine whether this recombinant protein could be purified for further biochemical characterization, we proceeded further with the purification procedure using an affinity matrix adapted to the *Strep*-tag II (Figure 4B). As the recombinant ceQORH protein could be released from the membrane without detergent, we chose to solubilize it using a 1M NaCl treatment for further purification steps. The solubilized membrane proteins were then desalted and further purified on a *Strep*-Tactin affinity matrix (Figure 4B). Interestingly, the protein was strongly enriched in the elution fractions (Figure 4B, lanes E3 and E4) and the yield of purification was 2–4 mg of purified protein per liter of culture. However, we noticed that some recombinant ceQORH protein was lost, since already eluted from the matrix using filtration and washing buffers (Figure 4B, lanes F and W).

### Solubilization and Purification of the Intrinsic Proteins AtHMA1 and AtHMA6

**Figure 5:**
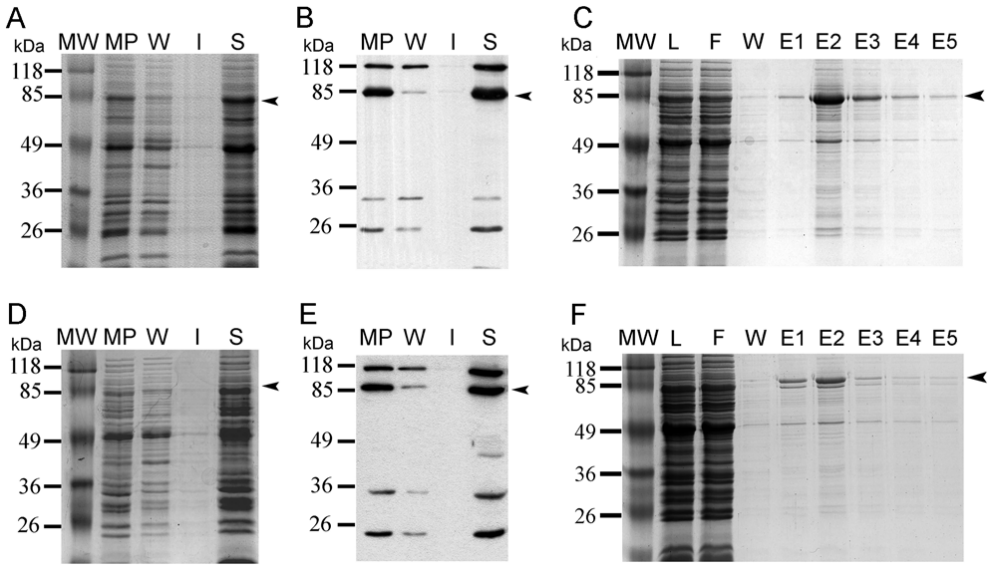
Solubilization and purification of two intrinsic recombinant AtHMA1 and AtHMA6 proteins. Panels A, B, D, E: Solubilization of AtHMA1 and AtHMA6 using detergents. Membrane proteins (MP, 4 µg/µL) were incubated in 50 mM Tris-HCl (pH 8.0), 100 mM NaCl and subsequently centrifuged to eliminate soluble proteins (W, washing). Membrane pellets were solubilized in the same buffer containing 1% DDM (w/v), 0.32% (w/v) C_12_E_8_ and 100 µM TCEP. After incubation for 1h30, solubilized membrane proteins (S) were separated from insoluble proteins (I) by centrifugation. Aliquots (15 µg of crude MP and 10 µL of resulting fractions W, I and S) were loaded on a 10% SDS-PAGE further stained with Coomassie blue (panels A, D) and by western blot (panels B, E) using the HRP conjugate specific to the *Strep*-tag II. Panels C and F: purification of AtHMA1 and AtHMA6, respectively, using a *Strep*-Tactin Sepharose matrix in a buffer containing 100 µM TCEP and 0.1% (w/v) DDM. Solubilized membrane proteins were loaded (L, 10 µL) on the column (F, 10 µL flowthrough). After washing the matrix (W, 10 µL), the bound proteins were eluted (E1 to E5, 20 µL) by addition of 2.5 mM desthiobiotin. Fractions were loaded on a 10% SDS-PAGE further stained with Coomassie blue. Arrows indicate the AtHMA1 (panels A, B, C) and the AtHMA6 (panels D, E, F) proteins.

AtHMA1 and AtHMA6 are intrinsic membrane proteins and their solubilization required the presence of detergents. Assays were first carried out using DDM and/or C_12_E_8_ (dodecyl octaethylene glycol monoether), which allowed the solubilization of AtHMA1 (Figure 5A–B) and AtHMA6 (Figure 5D–E) from *Lactococcus* membranes. Treatments with 1% (w/v) DDM only partially released these proteins in the fraction containing the solubilized membrane proteins (data not shown). To improve solubilization yields, sonication of the purified membrane fraction was performed in order to homogenize the suspension and to obtain smaller vesicles, more accessible to detergents. Sonication and addition of 0.32% (w/v) C_12_E_8_ allowed to almost completely solubilize the membrane proteins (Figures 5A–B and 5D–E, *lane S*). After treatment with 1% (w/v) DDM and 0.32% (w/v) C_12_E_8,_ solubilized AtHMA1 and AtHMA6 were purified on the *Strep*-Tactin affinity matrix. The purification was performed in a buffer containing 0.1% (w/v) DDM, and in the presence of 100 µM TCEP in order to stabilize the protein. Both AtHMA1 and AtHMA6 proteins were mainly recovered in the elution fractions and the yield of the purification process was approximately 30% for AtHMA1 and 10% for AtHMA6 (Figures 5C and 5F).

Taken together, the above-described results indicate that both peripheral and intrinsic plant membrane proteins can be solubilized and purified from crude membranes when expressed in *Lactococcus*.

### Functional Expression of the ceQORH and AtAATP1 Proteins Produced in *L. lactis* Cells

Trying to detect the activity of the recombinant HMA proteins, we performed ATPase assays on *L. lactis* membrane expressing the recombinant proteins AtHMA1 or AtHMA6. However, the endogenous ATPase activity of crude *L. lactis* membranes (from *L. lactis* strain transformed with a non recombinant plasmid) is quite high (see Figure S2). From experiments performed on the natural protein when present in the chloroplast envelope [22], the ATPase activity of AtHMA1 might only represent a few percent of the total endogenous ATPase activity measured in crude *L. lactis* membranes. We thus tried to detect the ATPase activity of the partially purified recombinant AtHMA1 and AtHMA6 proteins. At the present time, efforts to identify solubilization and purification conditions that are compatible with the detection (or the preservation?) of the ATPase activity associated to these purified proteins were not successful.

Based on sequence similarity, the *Arabidopsis* ceQORH protein belongs to the short-chain dehydrogenases/reductases (SDR) family [40]. SDRs are known to use artificial electron acceptors like nitroblue tetrazolium (NBT) as substrates, and activity of heterologously expressed and purified SDR requires conditions mimicking a lipid environment [41]–[43]. Since the native ceQORH protein is associated with the inner membrane of the chloroplast envelope [16], we tried to recover this dehydrogenase activity in combining the purified recombinant ceQORH protein with a soybean lipid extract as previously described [43]. Due to the low purification yield obtained with the ceQORH protein while tagged with a *Strep*-tag II (*C*-terminus), we constructed another vector, coding for the ceQORH protein with a *N*-terminal poly-histidine tag, as previously described for its expression in *E. coli* [16]. Using this construction, we were able to purify (solubilization with 1 M NaCl followed by affinity purification) sufficient amounts of the recombinant protein to analyze the shape of the ceQORH protein when produced in *L. lactis* (see Figure S3A). First, a gel filtration chromatography performed with this purified recombinant protein suggests that the recombinant ceQORH protein mostly behaves as a monomer with a low proportion of dimers and tetramers (Figure S4). We currently have no physiological explanation for the role of this partial oligomerisation *in vivo*. This new strategy, improving yield of purified ceQORH protein, was then used to further validate that the recombinant ceQORH protein is active when produced in *L. lactis*. Deshydrogenase assays were thus performed with NBT chloride as an artificial substrate, and the reaction (NBT reduction to formazan) was monitored using a spectrophotometer. As can be seen in the Table 1, the ceQORH protein, in combination with lipids and NADPH reduces the NBT to formazan. Heat-inactivation of the purified ceQORH protein totally abolished dehydrogenase activity (data not shown). Similarly, no residual activity was detected in assays lacking the ceQORH protein (Table 1). A low activity (10%) was detected in the absence of lipids and some lower residual activity (6%) was also detected in assays lacking NBT (Table 1). Altogether, these data clearly demonstrate that the recombinant ceQORH protein is active when produced in *L. lactis*. It is very hard to estimate the percentage of active recombinant ceQORH protein as this could only be done using comparison of its specific activity with the one of the purified natural plant protein. When combining the low amount of the ceQORH protein within the chloroplast envelope (approximately 1% of the envelope proteins) [16] with the low amount of envelope proteins within a chloroplast (approximately 1% of the chloroplast proteins), purification of the natural protein is almost unreachable. As an alternative to the comparison of the recombinant ceQORH protein with its natural counterpart, we choose to compare the activity of the recombinant ceQORH protein produced in *L. lactis* with the same ceQORH protein produced in *E. coli*. While mostly recovered as inclusion bodies when produced in *E. coli* [16], few percent of the produced recombinant ceQORH protein could be purified from the bacterial extract (see Figure S3A). The specific activity of this protein was then compared to the activity of the same protein expressed and purified from *L. lactis*. As presented in table 1B, the ceQORH protein produced in *L. lactis* has a higher specific activity when compared to the protein produced in *E. coli*.

**Table 1:** Specific deshydrogenase activity of the recombinant ceQORH protein.

**A. Deshydrogenase activity of the ceQORH protein produced in ** ***L. lactis***	
**System**	**Specific activity of the ceQORH protein**
Complete	19,28 (100%)
Without ceQORH	0 (0%)
Without PC	1,97 (10,2%)
Without NADPH	0 (0%)
Without NBT	1,14 (5,9%)
**B. Comparison with other forms of the recombinant ceQORH protein**	
ceQORH protein produced in *E. coli*	40%
ceQORHatt protein produced in *L. lactis*	95%
ceQORHnh protein produced in *L. lactis*	90%

**A**. The deshydrogenase activity of the purified recombinant HisceQORH proteins was measured as described in materials and methods. The impact of the lack of every reaction components was tested. The specific activity determined for the HisceQORH protein (100%) was 19.3 nmoles of formazan mg/ceQORH protein/min. **B**. Dehydrogenase activity of the ceQORHnh protein and of the ceQORHatt protein (containing the *N*-terminal “att” extension) produced in *L. lactis* and the ceQORH protein produced in *E. coli*.

As described above, addition of an *N*-terminus coded by the *att*B sites to the ceQORH protein has no impact on the production of this recombinant protein when produced in *L. lactis* (figure 3A). We also questioned the effect of this *N*-terminal fusion (coded by the *att* sites) on the activity of the recombinant ceQORH protein. The two forms (with or without the corresponding *N*-terminal extension) of the recombinant ceQORH protein were thus produced in *L. lactis* and purified (see Figure S3B). Dehydrogenase assays, carried out on the recombinant proteins lacking (ceQORH) or containing this *N*-terminal extension (ceQORHatt), also demonstrated that the specific activities of these two proteins are similar (table 1B).

**Figure 6:**
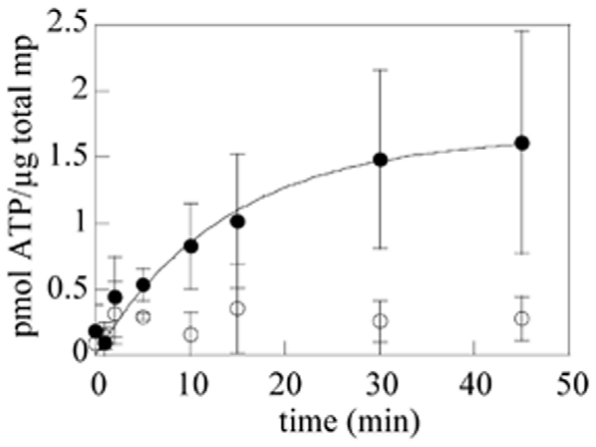
Transport kinetics (ATP uptake) of the recombinant AtAATP1 protein in *L. lactis*. After induction of expression with nisin for 4 h at 30°C, *L. lactis* cells expressing the AtAATP1 protein or *L. lactis* cells containing the empty vector (as a negative control), were incubated with 50 µM [α-^32^P] ATP for the indicated time periods. Data are the mean of four independent experiments and standard error to the mean are displayed. The graph shows the difference of ATP transport efficiency (expressed in pmol ATP/µg total membrane proteins) between *L. lactis* cells expressing the AtAATP1 protein (full circles) or *L. lactis* cells containing the empty vector (empty circles).

The AtAATP1 protein was previously demonstrated to transport ATP when expressed in *E. coli* [44]. In order to validate that, when expressed in *L. lactis*, the recombinant AtAATP1 protein was also active, we performed similar experiments based on uptake of radioactive ATP. Using intact *L. lactis* cells expressing the recombinant AtAATP1 protein, we could measure a time dependence uptake of [α-^32^P] ATP into bacteria (Figure 6). This uptake of radiolabeled ATP increased with time up to 1.5 nmol ATP/mg total *L. lactis* membrane proteins (Figure 6). It is noteworthy *i*) that the uptake is linear in the first few minutes and then reaches a stationary phase and *ii*) that no further ATP uptake was detectable after 30 min of reaction. In contrast, and as a negative control, *L. lactis* cells carrying the empty vector did not import radiolabeled ATP at a significant rate. This result indicates that the recombinant AtAATP1 protein produced in *L. lactis* is able to transport ATP, and is thus produced in an active form.

## Discussion

Like many other groups aiming to perform the biochemical characterization of membrane proteins, we were strongly limited by the absence of production obtained for our favorite *Arabidopsis* membrane proteins using traditional expression systems. The aim of this work was thus to test the expression, in *L. lactis*, of peripheral and intrinsic *Arabidopsis* membrane proteins that could not be produced using more classical heterologous expression systems.

### A Cloning Strategy Compatible with Gateway-Entry Vectors to Express Proteins in *Lactococcus*

This article first describes a cloning strategy based on the NICE system to facilitate cDNA transfer from Gateway entry-vectors into a nisin-inducible vector of *L. lactis.* As the nisin-inducible vector pNZ8148 could not be converted into Gateway destination vector, we elaborated this strategy, which relies on simple steps: PCR, Gateway LR reaction and endonuclease digestion (Figure 1). Every Gateway vector possesses *att* sites necessary for recombination and easy cloning of cDNA. We thus checked whether the presence of these sequences affects the expression level of the recombinant proteins. The presence of these *att*B sites between the ATG of the *Nco*I site and the ATG of the cDNA had no effect on the level of production (see Figure 3) when comparing constructs elaborated with the present strategy and a classical strategy [14]. Furthermore, it is well documented that codon usage is one of the main factors interfering with efficient production of eukaryotic proteins in microorganisms [13]. Various options have been proposed to improve protein expression when codon usage is limiting. In some cases, gene synthesis with optimization of the codon usage of either the whole cDNA or its 5′ end [45], [46] proved to be efficient means in order to increase the yield of protein production. Using the present strategy, the first 17 codons of every expressed protein are identical thus avoiding the use of expensive and time-consuming strategies to optimize codon usage in the *N*-terminus of proteins.

In 2007, Geertsma and Poolman [47] have described a strategy for high-throughput cloning and expression in bacteria. This strategy involved ligation-independent cloning in an intermediary *Escherichia coli* vector, which is rapidly converted via vector-backbone exchange (VBEx) into an organism-specific plasmid ready for high-efficiency transformation. Another advantage of the strategy developed by Geertsma is that it allows directional cloning of the cDNAs. Such a protocol allows cloning of a cDNA into a *Lactococcus* vector into four days. The cloning strategy described here in principle is completely compatible with the VBEx method. The present strategy requires only three days, but is not as generic as the VBEX method because of the requirement of the *Eco*RV digestion. However, the present strategy should be a useful tool for laboratories that use the Gateway system to clone their cDNAs of interest in allowing direct transfer of these constructions into a *Lactococcus* expression vector. Using the present cloning strategy, we successfully produced proteins from *Arabidopsis* but also proteins from both prokaryotic and eukaryotic kingdoms whatever their hydrophobicity or their size (unpublished results).

### The ceQORH Protein Expressed in *Lactococcus* Is Functional and Behaves as the Native Protein

*L. lactis* is a Gram-positive bacterium, which presents several advantages when compared to *E. coli.* One of the major advantages of *L. lactis* relies on the fact that it does not form inclusion bodies [48]. When expressed in *E. coli*, the ceQORH protein was mainly recovered as inclusion bodies [16] and only 10% of the expressed protein could be used for biochemical analysis. In *L. lactis*, this protein was recovered in the membrane fraction and represented 20 to 30% of total membrane proteins, a relatively high percentage close to levels of expression obtained for prokaryotic membrane proteins produced in *L. lactis* [14].

Solubilization of the ceQORH protein was performed as previously done for the native protein in its native membrane [16] led to similar results in *Lactococcus*. The ceQORH protein produced in *L. lactis* thus behaves as the natural chloroplast envelope protein and appears to interact with the bacterial membrane through electrostatic interactions. This interaction with membranes was not observed for the recombinant protein expressed in *E. coli* [16]. Junge and coworkers [15], and Opekarova and Tanner [42] underlined the importance of the lipid composition of host cells in overexpression of functional membrane proteins. Therefore, one possible explanation might derive from the *Lactococcus* membrane composition, which is much closer, to the one of the inner membrane of the chloroplast envelope. Indeed, *L. lactis* membrane contains glycolipids [49] like the inner membrane of the chloroplast envelope [50]. In contrast, *E. coli* membranes do not contain any glycolipids [51].

The presence of two forms of ceQORH proteins (see Figure 4A) was surprising. These two forms of the protein certainly hold the same *C*-terminus, since both forms are recognized by the conjugate directed against the *Strep*-tag II, and since this tag is fused at this extremity of the protein. The difference in size might thus be explained by the presence of several ATG initiation codons in the 5′ of the cDNA sequence. These initiation codons are in frame with the ATG initiation codon present in the vector (within the *Nco*I restriction site, see Figure 1B). The first ATG in the ceQORH cDNA is already a second potential initiation codon (+1), but two other ATG codons are also present at +6 and +56 positions. The presence of Shine Dalgarno-like sequences in 5′ of these ATG might create several independent sites of translation initiation of the genes leading to the production of two proteins different in size (2-3 kDa). Alternatively, proteolysis of the *N*-terminal extremity of the recombinant protein might also occur resulting in the presence of a shorter form of the recombinant protein.

The ceQORH protein is structurally related to bacterial, fungal, and animal proteins of known quinone oxidoreductase function. During earlier work, Jäger-Vottero and coworkers [52] presented spectroscopic evidence for the presence of electron carriers (redox chains) in chloroplast envelope membranes. They also detected an NADPH quinone oxidoreductase activity in the chloroplast envelope, presumably associated to these redox chains. This activity may thus result from the presence of the ceQORH protein. Using the ceQORH protein produced in *L. lactis*, we were able to establish that the ceQORH protein indeed has NADPH-dependent dehydrogenase activity, and that this activity requires a lipid environment. The physiological role of the ceQORH protein and the significance of these redox chains remain to be determined. However, the production of large amounts of recombinant ceQORH protein now opens the way to further enzymatic assays that will be performed searching for native chloroplast envelope molecules in order to determine the genuine substrate of the ceQORH protein.

### Expression of Intrinsic Proteins of the P-Type ATPase Family in *L. lactis*

AtHMA1, 3, 4 and 6 are four proteins belonging to the P-type ATPase family. These proteins are intrinsic membrane proteins with several (6 to 8) predicted transmembrane domains. AtHMA3 was found to be a vacuolar transporter whose overexpression in plant results in Cd, Pb and Zn tolerance [23]. AtHMA4 is localized in the plasma membrane and is implicated in Zn homeostasis and Cd tolerance [53], [54]. Both proteins were characterized using mutant plants and by functional expression in yeast or functional complementation of yeast mutant strains. However, this eukaryotic heterologous system does not allow the production in sufficient amount of these proteins for further biochemical and structural analyses. AtHMA6 is associated with the chloroplast envelope and is involved in copper uptake into the chloroplast. The function of this protein was deduced from the analysis of the phenotype of corresponding mutant plants [21] but, to date, the biochemical characterization of this protein have not been reported in the literature. AtHMA1 is another ATPase of the chloroplast envelope which has been implicated in Cu, Zn and Ca transport from functional expression in yeast or *in planta* analyses [22], [55], [56]. The main limit of all these studies results from huge difficulties encountered by biochemists to produce highly hydrophobic membrane proteins in sufficient amount for direct characterization of their specificity. It was thus essential to produce AtHMA1 in a heterologous system, compatible with biochemical characterization of the protein, to get more insights into the ionic specificity of this ATPase.

To date, only the plant ATPase AtHMA2 has been functionally characterized after heterologous expression in yeast (ion selectivity, activity rate, direction of transport, catalytic phosphorylation, etc. [57]), and only its *C*-terminal metal binding domains have been expressed in a prokaryotic system. The unsuccessful expression of these proteins in *E. coli* is probably due to their toxicity when expressed in bacterial cells. The toxicity could result from the presence of elevated amounts of proteins at the membrane and/or from the impact of the ATPases on the homeostasis of cations within the cell. During this work, we successfully expressed four *Arabidopsis* P_1B_-type ATPases in *Lactococcus* with the NICE system thus suggesting that *L. lactis* is able to deal with the production of potentially toxic plant proteins.

To the best of our knowledge, this article is the first report of successful expression in a prokaryotic host, solubilization and purification of plant ATPases. The obtained yield of production (1–3% of total membrane proteins, except for AtHMA4 which represent only 0.2% of total membrane proteins), is relatively high when compared to other eukaryotic membrane proteins already produced with the same expression system [14]. Indeed, from 1 liter of culture, we obtained 30 mg of total membrane proteins containing 300 to 900 µg of the produced recombinant protein. These amounts are thus compatible with biochemical assays on total membrane extracts, but also with further solubilization and purifications steps.

### Solubilization/Purification of P-Type ATPases Produced in *L. lactis*

During this work, we successfully expressed AtHMA1 and AtHMA6 and the next step was the solubilization of these intrinsic membrane proteins. They belong to the P-type ATPase subfamily whose catalytic mechanism is similar to the well-characterized P-type enzymes such as the sarcoplasmic Ca-ATPase (SERCA) [18]. The crystal structure of the SERCA1A has been solved [58], [59] and the detergents used for solubilization were either DDM and/or C_12_E_8_ [59], [60]. DDM has been successfully used for solubilization of membrane proteins [61] and its efficiency could be explained by its polar headgroup structure [62]; C_12_E_8_ has been demonstrated not to affect SERCA integrity during solubilization [59]. Solubilization trials were performed with these two detergents and led to an almost complete solubilization of the proteins.

Some members of the P-type ATPase family such as SERCA and AtHMA2 have already been solubilized and/or purified in buffers containing a low percentage of DDM and TCEP [31], [59], [60]. TCEP is a reducing agent, which presents several advantages when compared to DTT: TCEP is stable at either acidic pH or above pH 7.5 and is suitable for storage of purified proteins [63]. This compound did not alter the proper function of AtHMA2 [31]. Purification of both AtHMA1 and AtHMA6 proteins on *Strep*-Tactin matrix was performed in the presence of 0.1% (w/v) DDM and 100 µM TCEP and resulted in a purification yield of around 10 to 30%. Biochemical characterization of these proteins can now be considered as well as structure/function studies. These analyses should provide additional information on the substrates specificity of these ATPases and on the role of conserved residues in the catalytic activity of the proteins. Furthermore, yields of expressed protein are also compatible with crystallization trials.

### Expression of Active AtAATP1 in *L. lactis*

The import of ATP from the cytoplasm is essential for many of the metabolic functions carried out within plastids. This import is mediated by specific ATP/ADP transporters, which import ATP in exchange with ADP. AtAATP1 is one of the adenylate translocators identified in the chloroplast and this transporter has already been functionally characterized after expression in *Saccharomyces cerevisiae* and *E. coli* cells [26], [44].

We successfully expressed the AtAATP1 protein in *L. lactis* with a yield of production around 0.2% of total membrane protein. Despite this low expression, *L. lactis* seems to be a very efficient heterologous system to produce the AtAATP1 protein. During this work, uptake assays of radioactive nucleotides has been performed and showed a time dependent uptake of ATP. This indicates that, in *L. lactis*, the produced protein is correctly folded and able to transport radioactive ATP into cells with the similar mechanism observed in chloroplasts, its original organelle. Interestingly, the rate of ATP uptake in *L. lactis* (0.5 nmol ATP/mg protein for 2 minutes) was similar to the one measured in *E. coli* cells (0.66 nmol ATP/mg protein for 2 minutes) [44].

### *L. lactis,* an Alternative System for Functional Expression of Peripheral and Intrinsic *Arabidopsis* Membrane Proteins

*Lactococcus lactis* is a recognized tool for the expression and study of integral membrane proteins of both prokaryotes and eukaryotes [14], [48], [64]. The NICE system has already been used for expression of soluble plant proteins, like the *Arabidopsis* coumarate:CoA ligase [8] and the strawberry alcohol acyltransferase and linalool/nerolidol synthase [65]. However, and to our knowledge, the present study is the first report of the expression of plant membrane proteins with the nisin-inducible expression system of *L. lactis*. Mierau and Kleerebezem [13] explained that, the more closed the GC content is to *L. lactis*, the higher the probability of the gene to be successfully expressed is. The relatively high production yield of the *Arabidopsis* membrane proteins might thus result from a similar GC content in both *L. lactis* (35%; [66]) and *Arabidopsis thaliana* (36%; [67]). The ceQORH protein can also be produced in *E. coli* with the same level of production. However, in *E. coli* more than 90% of the protein is recovered as inclusion bodies [16]. The intrinsic protein AtAATP1 was also expressed and functional in *E. coli* system but the level of production in this system might be lower than the one obtained in *L. Lactis* system [44]. Indeed, the recombinant AtAATP1 protein could only be detected thanks to the use of radioactively labeled methionine while, in *Lactococcus*, the recombinant protein is detected with the Strep-tactin conjugate. Functionnal characterization of the AtHMA1,3,4 proteins was performed in yeast system and also in *E. coli* for AtHMA4 [22], [68], [69]. However, none of these studies relates the production level of these proteins and the corresponding recombinant proteins were not used for further biochemical analysis.

To conclude, *L. lactis* appears to be an appropriate expression system for *Arabidopsis* membrane proteins. This system offers new possibilities, for the characterization of intrinsic and low abundance plant proteins, which cannot be achieved using the natural proteins or other less-adapted heterologous systems.

## Materials and Methods

### Bacterial Strains and Growth Conditions

The *L. lactis* and *E. coli* strains used in this study are listed in Table 2. Lactococcal strains were grown in M17 medium (Difco, USA; [70]) supplemented either with 0.5% (w/v) glucose (M17G medium) at 30°C without shaking for DNA isolation or with 1% (w/v) glucose (M17G1) at 30°C with gentle shaking (90 rpm) for expression of recombinant proteins. The bacterial growth is slightly higher with gentle shaking and the time required to reach an OD_600nm_ of 0.8 is shortened of about 30 min. *E. coli* strains were grown in Luria-Bertani (LB) medium at 37°C with shaking (200 rpm). Antibiotics were used for plasmid maintenance at the following concentrations: chloramphenicol (10 µg/mL) for *L. lactis,* ampicilline (100 µg/mL) and kanamycine (50 µg/mL) for *E. coli*.

**Table 2:** Bacterial strains and plasmids used in this study.

			Relevant genotype or phenotype	Reference(s) or sources	
**Strains**					
	*E. coli*				
		Top 10	F- mcrA ϕ80lacZΔM15 ΔlacX74 recA1 araD139	Gateway, Invitrogen	
			galU galK rpsL (StrR) endA1 nupG		
	*L. lactis*				
		NZ9000	MG1363 *pepN*::*nisRK*	NIZO	
		NZ9700	Progeny of the conjugation between nisin producer strain NIZO B8 with MG1614 (Rif^R^ Strp^R^ derivative of MG1363). Nisin producer strain for induction experiments	NIZO	
**Plasmids**					
	pBlueScript-SK+		Kan^r^	Fermentas	
	pENTR-D-TOPO		attL1 and attL2 sites; Kan^r^	Gateway, Invitrogen	
	pDONR221		attP1 and attP2 sites; Kan^r^	Gateway, Invitrogen	
	pBS-RfA		attR1 and attR2; Amp^r^	This work	
	pNZ8148		Cm^r^	NIZO	

Kan^r^, Amp^r^ and Cm^r^: resistance to kanamycine, ampicilline and chloramphenicol, respectively.

### Oligonucleotides

The different primers used to amplify the cDNAs introduced into Gateway entry vector or into pNZ8148 are listed in Table 3.

**Table 3:** Oligonucleotides primers used for Gateway and classical cloning strategies.

**A. Oligonucleotides primers used for Gateway cloning strategy**		
Protein	Oligonucleotides	Sequence 5′- 3′
ceQORH	ceQORH-TOPO fwd	CACC***ATG*** *GCTGGAAAACTCATGCACGC*
	ceQORH-TOPO rev	***CTA*** CTTTTCGAACTGCGGGTGGCTCCA*TGGCTCGACAATGATCTTCCCAG*
AtHMA1	AtHMA1-TOPO fwd	CACC***ATG*** *CTACGTGCTGTCGAAGATC*
	AtHMA1-TOPO rev	***CTA*** CTTTTCGAACTGCGGGTGGCTCCA *ATGTGCAGAGCTTAAACTGTTG*
AATP1	AATP1-attB1 fwd	GGGGACAAGTTTGTACAAAAAAGCAGGCTCC***ATG*** *GCGGAGGCCGCGGCTGCT*
	AATP1-attB2 rev	GGGGACCACTTTGTACAAGAAAGCTGGGTC***CTA*** CTTCTCGAATTGTGGGTGTGACCA *TAAGTTGGTGGG*
**B. Oligonucleotides primers used for classical cloning strategy**		
Protein	Oligonucleotides	Sequence 5′- 3′
ceQORH	ceQORH rev	*GTTAGACCCGCCACAGGTAA*
	ceQORHNcoI fwd	CATG**CC** ***ATG*** **G** *CTGGAAAACTCATGCAC*
	ceQORHHindIII rev	CCC**AAGCTT** ***CTA*** CTTTTCGAACTGCGGGTGGCTCCA *TGGCTCGACAATGATCTTCCCAG*
AtHMA1	AtHMA1 rev	*GACTTCCCCAGTCAAGTGCT*
	AtHMA1NcoI fwd	CATG**CCATGG**CC***ATG***C*TACGTGCTGTCGAAGATC*
	AtHMA1HindIIIrev	CCC**AAGCTT** ***CTA*** CTTTTCGAACTGCGGGTGGCTCCA *ATGTGCAGAGCTTAAACTGTT*G
AtHMA3	AtHMA3 rev	*CAAGAATGGGGAAAACTCCA*
	AtHMA3NcoI fwd	CATG**CC** ***ATG*** **G** *CGGAAGGTGAAGAG*
	AtHMA3TEV rev	GCGGGTGGCTCCA TCCCTGAAAATACAGGTTTTC*CTTTTGTTGATTGTC*
AtHMA4	AtHMA4 rev	*GCCAACGTAAAGGCGAGTAG*
	AtHMA4NcoI fwd	CATG**CC** ***ATG*** **G** *CGTTACAAAACAAA*
	AtHMA4TEV rev	GCGGGTGGCTCCA TCCCTGAAAATACAGGTTTTC*AGCACTCACATGGTG*
	AtHMAKpnI rev	CGG**GGTACC** ***CTA*** CTTTTCGAACTGCGGGTGGCTCCATCCCTGAAAATACAG
PAA1	PAA1 rev	*CTCGAGGAGTAGACTGAAAC*
	PAA1NcoI fwd	CATG**CCATGG**CC***ATG*** *AGTGGCGGCGGTTCTGG*
	PAA1HindIII rev	CCC**AAGCTT** ***CTA*** CTTTTCGAACTGCGGGTGGCTCCA *AGAGCTTTGCTTCCATC*
AATP1	AATP1 rev	*GCCCCAGAAGAGAACTGAGA*
Vector	pNZ8148_fwd	CGCGAGCATAATAAACGGCTCTG

The start (ATG) and stop (CTA) codons are indicated in bold italics. The coding sequences of the cDNAs are indicated in italics. The sequence coding for the *Strep*-tag II is underlined. The restriction sites (*Nco*I, *Hind*III and *Kpn*I) are indicated in bold. The *att*B sites (in A) and the sequence coding for the TEV protease site (in B) are highlight in grey.

### Accession Numbers

The AGI (Arabidopsis Genome Initiative) or TrEMBL accession numbers corresponding to gene loci on the *Arabidopsis* genome or to the entries of the proteins, respectively, are listed for each protein: ceQORH (At4g13010; Q9SV68), AtHMA1 (At4g37270; Q9M3H5), AtHMA3 (At4g30120; Q9SZW5), AtHMA4 (At2g19110; Q66474), AtHMA6 (At4g33520; Q9SZC9) and AtAATP1 (At1g80300; Q39002). Three of these plant proteins are known to be processed during their import into the chloroplast envelope. When compared to the original primary sequence deduced from the corresponding cDNAs, production of the mature forms of these proteins thus required the deletion of the first 60, 79 and 102 amino acid residues corresponding respectively to the chloroplast transit sequences of AtHMA1 [22], AtAATP1 [27], [28] and AtHMA6 [21]. The cloned cDNA sequences code for the mature forms of these three proteins.

### DNA Techniques

Plasmid DNA extraction and ligation reactions were performed as previously described [71]. Restriction endonucleases (NEB, USA) and modification enzymes (Roche) were used according to the manufacturers' instructions. PCR amplifications were performed according to the protocol provided with the amplification kit (Phusion High-Fidelity DNA Polymerase, Finnzymes, Finland).

### Cloning into Gateway Entry Vectors

Plasmids used in this study are listed in Table 3A. The two cDNAs (coding for ceQORH and AtHMA1) were amplified by PCR from already available recombinant plasmids in one step using corresponding primers (Table 3B). AtAATP1 cDNA (pda01438) was obtained from RIKEN BioResource Center (BRC) and amplified by PCR using the “AATP1-attB1 fwd” and “AATP1-attB2 rev” primers (Table 3B). The three PCR fragments were inserted into the entry vector pENTR-D-Topo (ceQORH, AtHMA1) and pDONR221 (AtAATP1). Subsequently, the plasmid pBlueScript (Fermentas) has been converted by the Gateway® Vector Conversion System (Invitrogen) into a destination vector by insertion of an RfA cassette (Invitrogen) in order to maintain the correct reading frame, leading to the generation of the plasmid pBS-RfA. This RfA cassette is surrounded by two *Eco*RV sites for further excision of the whole cassette. A LR reaction was performed according to the manufacturer instructions (Invitrogen). This reaction allowed the transfer of the cDNA from the entry vector to pBS-RfA and the generation of the “shuttle” vector pBS-RfA-cDNA.

### Cloning cDNAs from Gateway Entry Vectors into pNZ8148NK

The nisin-inducible vector pNZ8148 (NIZO, The Netherlands; Table 2) is an improved version of pNZ8048 with a deletion of 60 bp residual DNA from *Bacillus subtilis* corresponding to the initial cloning host of the pSH series of plasmids [13]. It contains the P_nisA_ promoter, a multicloning site (*Nco*I, *Pst*I, *Sph*I, *Kpn*I, *Spe*I, *Xba*I, *Sac*I, *Hind*III) and the *Nco*I site containing the ATG site in frame with the promoter for translational fusions (Figure 1A; see also the web site “Mobitec”: http://www.mobitec.de/de/products/bio/04_vector_sys/index.php?nisin.html). pNZ8048 has been constructed from an ancestor vector termed pNZ124 carrying the gene for the chloramphenicol resistance and the pSH replicon.

The three independent cDNAs were excised from the pBS-RfA-cDNA vector by digestion with *Eco*RV and ligated into pNZ8148NK. pNZ8148NK was obtained by digestion with *Nco*I and subsequent treatment by Klenow fragment (Roche, Switzerland) which filled the cohesive ends. When possible, a positive selection of recombinant vectors was obtained using digestion of the ligation products with *Nsi*I, a restriction site that was formed by religation of empty vector. Afterwards, the digested ligation product was purified, eluted in water and used to transform NZ9000 strain by electroporation [72]. Chloramphenicol-resistant bacterial clones were selected on M17GChl Agar Petri dishes after 1-2 days at 30°C. Right orientation of the expression cassette containing the cDNA and correct sequence of the clones were then confirmed by *i*) digestion of the plasmids extracted from bacterial cultures, *ii*) PCR amplification using a combination of the “pNZ8148 fwd” forward primer and of a reverse primer specific for each cDNA (Table 3B), and *iii*) sequencing analysis. Finally, a glycerol stock (15% (v/v)) was prepared for each recombinant bacterium containing the right clones and kept at −80°C.

### Direct Cloning of ceQORH and AtHMA1 into pNZ8148NH Vector

The two cDNAs were amplified by PCR, from already available plasmids, using the corresponding primers (Table 3B) to introduce new *Nco*I and *Hind*III restriction sites. PCR fragments were purified using the Nucleospin Extract II kit (Macherey Nagel, Germany). They were then digested with the endonucleases, *Nco*I and *Hind*III, by partial digestion [71] due to the presence of these two sites in both cDNAs. Subsequently, the digested fragments were ligated into pNZ8148NH previously digested with the same endonucleases. The two ligation reactions were purified, eluted in water and then used to transform NZ9000 strain by electroporation [72] Chloramphenicol-resistant clones were selected on M17GChl Agar Petri dishes after 1–2 days at 30°C. Presence of the cDNA and correct sequence of the clones were confirmed by both endonuclease digestion and sequencing analysis. The two corresponding vectors were termed pNZ8148NH-ceQORH and pNZ8148NH-AtHMA1.

### Construction of AtHMA6 Expression Vector

cDNA (pda19661) was obtained from RIKEN BioResource Center (BRC) and amplified by PCR using the “AtHMA6NcoI fwd” and “AtHMA6HindIII rev” primers (Table 3B). Indeed, the presence of several *Eco*RV sites within the cDNA coding for AtHMA6 prevented us from using the Gateway-compatible strategy. The PCR fragment was then digested with *Nco*I and *Hind*III and cloned into pNZ8148NH.

### Construction of AtHMA3 and AtHMA4 Expression Vectors

cDNA were amplified by PCR from already available recombinant plasmids, in two steps, using corresponding primers (Table 3B). The first PCR step was performed using the primers containing the *Nco*I site (forward) and the TEV protease site (reverse), thus allowing insertion of this protease site for further cleavage of the *Strep*-tag II from the purified proteins. The second PCR step, performed with primers containing the *Nco*I site (forward) and the *Strep*-tag II and *Kpn*I site (reverse) inserted the *Strep*-tag II sequence downstream the TEV protease site. The PCR fragment was then digested with *Nco*I and *Kpn*I and cloned into pNZ8148NKpnI.

### Preparation and Storage of Nisin

M17G1 medium (10 mL) was inoculated with a concentrated glycerol stock of the *Lactococcus* NZ9700 strain and incubated for 6 hours at 30°C with gentle shaking (90 rpm). This preculture was then used to inoculate 500 mL of M17G1 medium followed by an overnight incubation at 30°C with gentle shaking (90 rpm). Finally, after a centrifugation at 6350 *g* for 5 min, at 4°C, the supernatant containing the nisin was stored at −80°C.

### Expression of Recombinant Proteins

Precultures (25 mL of M17G1Chl medium containing 10 µg/mL chloramphenicol) were inoculated with concentrated glycerol stock of recombinant bacteria and incubated overnight at 30°C with gentle shaking (90 rpm). These precultures were then added to 1 L of M17G1Chl in Schott bottles and incubated at 30°C with gentle shaking until the OD_600_ reached 0.8. Expression of target proteins was induced by nisin (see Results part for dilution used; for every round of nisin production, systematic tests were performed to determine optimal dilutions required for production of target proteins) and cultures were incubated for four additional hours. Bacteria were harvested by centrifugation at 5000 *g* for 15 min, at 4°C. The bacterial pellets were resuspended in 40 mL Tris-HCl 50 mM (pH 8.0), 100 mM NaCl and kept at −20°C overnight.

### Purification of Crude Bacterial Membrane Proteins

French Press was used for disruption of cells expressing the ceQORH protein. After addition of lysozyme (final concentration of 10 mg/mL), the bacteria were incubated at 30°C for 30 min to allow digestion of peptidoglycan. Subsequently, they were ruptured by 3-fold passages through a French Press cell at 18,000 p.s.i. (1.15 kbars) (French® Pressure Cell Press, SIM-AMINCO, Spectronic Instruments, Rochester, NY) and kept on ice until centrifugation. The cell disruptor was used for cells expressing the five intrinsic membrane proteins. The bacteria were disrupted by 2-fold passages through a One Shot (Constant Cell Disruption Systems, Northants, UK) at 35,000 p.s.i. (2.3 kbars) and kept on ice until centrifugation.

After cell breakage, the lysates were centrifuged at 10,000 *g* for 10 min, at 4°C, and the supernatant containing the proteins was transferred into Ti45 tubes for further ultracentrifugation at 150,000 *g* for 1 h, at 4°C. Membrane proteins present in the pellets were resuspended in 2 to 5 mL of 50 mM Tris-HCl (pH 8.0), 100 mM NaCl/Glycerol 20%.

### SDS-Polyacrylamide Gel Electrophoresis and Western Blotting

Protein content of membrane fractions were estimated using the Bio-Rad protein assay reagent (Bio-Rad, Hercules, CA; [73]). SDS-PAGE analyses were performed as described by Chua [74]. The recombinant *Strep*-tag II fusion protein (MW ∼28 kDa; 0.1 µg/µL; IBA, Goettingen, Germany) was used as a positive detection control and to estimate the amount of recombinant protein produced. Western blot were performed with the *Strep*-Tactin HRP conjugate (IBA, Goettingen, Germany) at a 1/10,000 dilution, followed by an ECL detection. Due to the low expression of the AtAATP1, immunoblotting has been adapted from Witte and coworkers [75]. Membranes were first blocked in TBS-blocking buffer (TBS buffer with 0.05% (v/v) Tween 20 and 4% (w/v) milk) for 1 h at room temperature followed by a second blocking step of 10 min in TBS-blocking buffer containing 40 µg/µL avidin and incubated with the *Strep*-Tactin HRP conjugate (1/4000 dilution) overnight at 4°C. The final washing steps consisted in three washes of 5 min in TBS-Tween buffer. The ceQORH protein was detected using the polyclonal antibodies raised against the recombinant *Arabidopsis* protein expressed in *E. coli* [16] at a 1/20,000 dilution followed by an ECL detection.

### Solubilization of the ceQORH Protein

Solubilization of the recombinant ceQORH protein present in the crude *Lactococcus* membrane proteins was performed as previously described for the natural protein present in chloroplast envelope membrane [16] with minor modifications. Membrane proteins (1 µg/µL) were incubated for 45 min, at 4°C, in 50 mM MOPS (pH 7.8) containing 0.5 or 1 M NaCl or 0.5 M KI. For alkaline treatments, membrane proteins were directly incubated in 0.1 M Na_2_CO_3_ (pH 11) or 0.1 M NaOH. For solubilization with detergents, membrane proteins were incubated in 50 mM Tris-HCl (pH 7.5) containing 0.1% (v/v) or 0.5% (v/v) of either Triton X-100 or n-dodecyl-β-maltoside (DDM, Sigma). Following treatments, membranes were centrifuged at 160,000 *g,* for 1 h, at 4°C to separate solubilized proteins from insoluble membrane proteins. Insoluble proteins were then resuspended in the same volume of 10 mM Tris-HCl (pH 6.8). Resulting fractions were then analyzed (20 µL) on a 12% SDS-PAGE and western blots were performed with either the polyclonal antibodies raised against the *Arabidopsis* ceQORH or the *Strep*-Tactin HRP conjugate.

### Solubilization of AtHMA1 and AtHMA6 Proteins

Membrane proteins (4 µg/µL) were incubated for 30 min at 4°C in 50 mM Tris-HCl (pH 8.0), 100 mM NaCl and subsequently centrifuged at 160,000 *g* for 80 min at 4°C to eliminate soluble proteins. Pellets were resuspended in the same buffer containing 20 mM DDM (1% (w/v)), 6 mM dodecyl octaethylene glycol monoether (C_12_E_8_, Sigma) (0.32% (w/v)) and 100 µM Tris(2-carboxyethyl)phosphine (TCEP, Sigma). Membranes were sonicated using a tipped sonicator for 3 min on ice (duty cycle = 10%, output control = 15). Membranes were then stirred gently for 1 h 30 at 4°C. After incubation, membranes were sonicated once more with the same settings. Membranes were then centrifuged at 15,000 *g*, for 20 min, at 4°C. Insoluble proteins were resuspended in 50 mM Tris-HCl (pH 8.0), 100 mM NaCl. Resulting fractions were then analyzed (10 µL) on a 10% SDS-PAGE and western blots were performed with the *Strep*-Tactin HRP conjugate.

### Purification of the ceQORH Protein

Membrane proteins solubilized with 1 M NaCl were desalted on a PD10 column (Sephadex G-25 M, GE Healthcare) and subsequently purified on *Strep*-Tactin Sepharose matrix (IBA, Goettingen, Germany) according to the manufacturer's instructions. The purified protein was then desalted on a PD10 column in 10 mM Tris-HCl (pH 8.0).

### Purification of AtHMA1 and AtHMA6 Proteins

Membrane proteins, solubilized with 1% (w/v) DDM and 0.32% (w/v) C_12_E_8_, were purified on *Strep*-Tactin Sepharose matrix (100 µL matrix/4 mg membrane proteins). The affinity matrix was equilibrated in purification buffer (50 mM Tris-HCl (pH 8.0), 100 mM NaCl, 100 µM TCEP, 0.1% (w/v) DDM) and then incubated with the solubilized membrane proteins for 1 h 30 at 4°C on a stirring wheel. This suspension was then loaded onto a micro spin column (BioRad 732–6204, Hercules, CA). The flow-through was stored for further analysis. The column was washed with 20 volumes (column bed volume) of purification buffer. The washing fraction was stored for further analysis. Targeted protein was eluted with 6×1 volume of purification buffer containing 2.5 mM desthiobiotin (IBA, Goettingen, Germany). An aliquot (20 µL) of each eluted fractions was analyzed on a 10% SDS-PAGE and western blots were performed with the *Strep*-Tactin HRP conjugate. Samples were stored at −80°C in 40% (v/v) glycerol.

### Deshydrogenase Activity Assay

Deshydrogenase assays with NBT chloride as substrate were adapted from [43]. In short, 1 µg of purified ceQORH was incubated with 1 µg of lipids (P3644, Sigma, Saint Louis, USA) at 30°C for 30 min before use in the enzyme assay. Deshydrogenase assays were carried out in 100 µL reactions containing 10 mM Tris-HCl (pH 8.0), 100 µM NADPH and 100 µM NBT. The reaction was started with addition of NADPH/NBT to the protein/lipids mixture. Formation of Formazan was followed at 550 nm on a Spectrophotometer (UV mc^2^, SAFAS Monaco) and the enzymatic activities were deduced from the OD measurement using the molar absorption coefficient [76].

### Uptake of Radioactively Labeled ATP

Uptake experiments were carried out according to Thuswaldner and coworkers [77] with a few modifications. Nisin-induced *L. lactis* (30 µL, 100 µg/µL) were incubated in 50 mM potassium phosphate buffer (pH 7.0) containing 50 µM [α-^32^P]ATP (3000 mCi/mmol; Perkin Elmer) at 25°C for indicated time periods. Uptake of nucleotides was quenched by addition of 1 mL of ice-cold potassium phosphate buffer. Subsequently, the cells were filtrated through a 0.45 µm filter (Millipore, France) under vacuum and washed three times with 1 mL of ice-cold potassium phosphate buffer. The radioactivity retained on the filters was quantified in 3.5 mL of water in a Multi-Purpose Scintillation Counter (Beckman Coulter, Fullerton, USA). The graphs have been generated using the KaleidaGraph version 4.02 (Synergy Software) and experimental data were fitted with a single exponential.

## Supporting Information

Figure S1 Quantification of the recombinant ceQORH protein in L. lactis membranes. L. lactis membrane proteins (MP, 5 and 10 µg) and various amounts of the purified recombinant ceQORH protein produced in E. coli (ceQORHrec, 0.3; 0.6; 1; 2; 3; 4 and 6 µg) were loaded on a 12% SDS-PAGE. Proteins were detected by Coomassie blue staining (upper panel, A) and the ceQORH protein was also detected by western blot (lower panel, B) using an anti-ceQORH polyclonal antibody (Miras et al., 2002). The arrow indicates the position of the ceQORH protein. Stars indicate similar amounts of the ceQORH protein. The estimated amount of the ceQORH protein in 10 µg of Lactococcus total membrane proteins (MP) is approximately 2 µg (western blot analysis) or 3 µg of recombinant protein (Coomassie blue staining). These data suggest that the recombinant ceQORH protein correspond to approximately 20 to 30% of the total membrane proteins from L. lactis.
(0.43 MB DOC)

Figure S2 ATPase activity of control (vv) L. lactis membranes or of L. lactis membranes containing HMA1 or HMA6. The ATPase assay mixture contained 250 mM Tris, pH 7, 15 mM MgSO4, 15 mM ATP, 20 mM Cysteine, 100 µM TCEP, 2 µM CuSO4, and 2 µg of membrane proteins. ATPase activity was followed for 10 min at 37°C. Released inorganic phosphate was colorimetrically determined (Lanzetta et al., 1979). Data are the mean of three independent experiments and standard error to the mean are displayed.
(0.27 MB DOC)

Figure S3 Analysis by SDS-PAGE of recombinant HisceQORH and Strep-tagged ceQORH proteins. A. Analysis by Coomassie blue-stained SDS-PAGE of the recombinant HisceQORH protein produced in L. lactis and E. coli. Increasing amounts of the two recombinant proteins (concentration range: 3, 6 and 9 µg) were loaded on the same gel. Note that the higher proportion of the ceQORH protein in protein extracts from L. lactis has an impact on the cleanness of the purified recombinant protein. B. Analysis by Coomassie blue-stained SDS-PAGE of the recombinant Strep-tagged ceQORH proteins (2 µg) produced in L. lactis without or with the N-ter extention (Ø att and att) encoded by the attB sites. As a control, increasing amounts of the recombinant His-tagged ceQORH protein (concentration range: 1, 2 and 4 µg) produced in L. lactis were loaded on the same gel.
(0.28 MB DOC)

Figure S4 Chromatographic separation of the ceQORH protein expressed in L. lactis. The recombinant ceQORH protein was first extracted from L. lactis membranes using a salt treatment (1 M NaCl) and then further purified using an affinity chromatography. The purified recombinant ceQORH protein (1.5 mg) was then loaded onto a Superdex 200 10/300 GL column (Pharmacia Biotech) in a buffer containing 20 mM Mops pH 7.8, 300 mM NaCl and 1 mM DTT. Calibration was performed (in the same buffer) using standard proteins from GE Healthcare (the gel filtration calibration kit contains Conalbumin 75 kDa, Ovalbumin 43 kDa, Carbonic anhydrase 29 kDa and Ribonuclease A 13.7 kDa). The ceQORH protein was recovered in three fractions (12.48 ml, 14.58 ml and 15.88 ml) corresponding to apparent molecular masses of 30 kDa, 55 kDa and 148 kDa. Knowing that the expected molecular mass of the monomeric form of ceQORH is 34 kDa (Miras et al., 2002), this result suggests that the recombinant ceQORH protein produced in L. lactis might behave, in presence of salt and DTT, as a mix of momoners, dimers and tetramers. The used calibration curve is presented in B.
(0.29 MB DOC)
