# Supplementary material for: Lactococcus lactis, an Alternative System for Functional Expression of Peripheral and Intrinsic Arabidopsis Membrane Proteins
Source: PLoS One. 2010 Jan 20;5(1):e8746. doi: 10.1371/journal.pone.0008746 (PMC2808337; doi:10.1371/journal.pone.0008746)
Supplement: Figure S1 — Quantification of the recombinant ceQORH protein in L. lactis membranes. L. lactis membrane proteins (MP, 5 and 10 µg) and various amounts of the purified recombinant ceQORH protein produced in E. coli (ceQORHrec, 0.3; 0.6; 1; 2; 3; 4 and 6 µg) were loaded on a 12% SDS-PAGE. Proteins were detected by Coomassie blue staining (upper panel, A) and the ceQORH protein was also detected by western blot (lower panel, B) using an anti-ceQORH polyclonal antibody (Miras et al., 2002). The arrow indicates the position of the ceQORH protein. Stars indicate similar amounts of the ceQORH protein. The estimated amount of the ceQORH protein in 10 µg of Lactococcus total membrane proteins (MP) is approximately 2 µg (western blot analysis) or 3 µg of recombinant protein (Coomassie blue staining). These data suggest that the recombinant ceQORH protein correspond to approximately 20 to 30% of the total membrane proteins from L. lactis. (0.43 MB DOC) [file pone.0008746.s001.doc]

**B**

**µg** 5 10 MW 0.3 0.6 1 2 3 4 6

**ceQORHrec**

**MP**

kDa

118

85

49

36

26

20


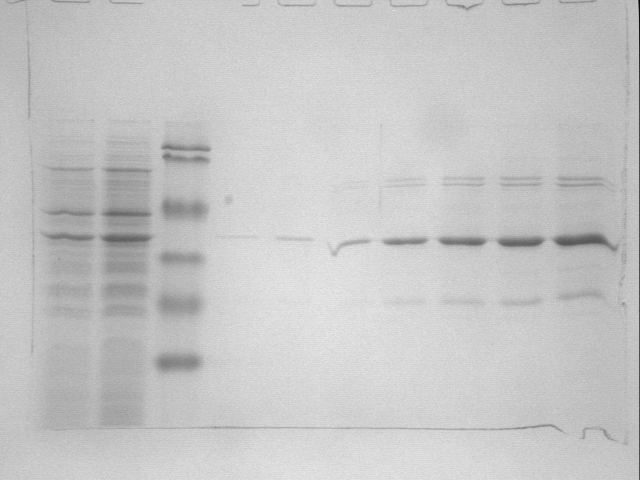


**A**

µg


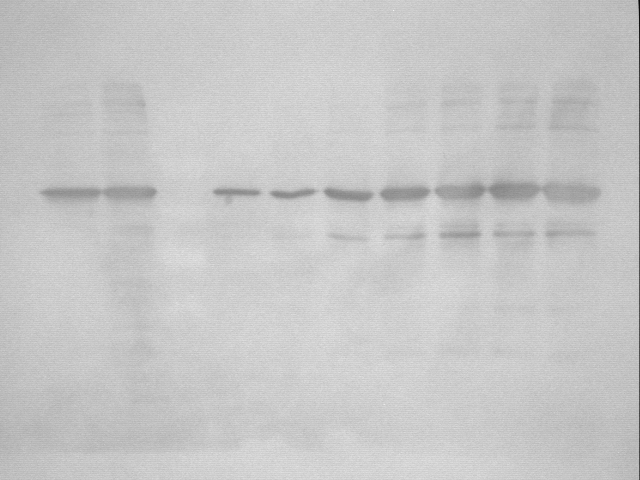


*

*

*

*

**Figure S1: Quantification of the recombinant ceQORH protein in *L. lactis* membranes.** *L. lactis* membrane proteins (MP, 5 and 10 µg) and various amounts of the purified recombinant ceQORH protein produced in *E. coli* (ceQORHrec, 0.3; 0.6; 1; 2; 3; 4 and 6 µg) were loaded on a 12% SDS-PAGE. Proteins were detected by Coomassie blue staining (upper panel, A) and the ceQORH protein was also detected by western blot (lower panel, B) using an anti-ceQORH polyclonal antibody (Miras *et al*., 2002). The arrow indicates the position of the ceQORH protein. Stars indicate similar amounts of the ceQORH protein. The estimated amount of the ceQORH protein in 10 µg of *Lactococcus* total membrane proteins (MP) is approximately 2 µg (western blot analysis) or 3 µg of recombinant protein (Coomassie blue staining). These data suggest that the recombinant ceQORH protein correspond to approximately 20 to 30% of the total membrane proteins from *L. lactis*.

Miras S, Salvi D, Ferro M, Grunwald D, Garin J et al. (2002) Non-canonical transit peptide for import into the chloroplast. J Biol Chem 277: 47770-47778
