# Supplementary material for: Lactococcus lactis, an Alternative System for Functional Expression of Peripheral and Intrinsic Arabidopsis Membrane Proteins
Source: PLoS One. 2010 Jan 20;5(1):e8746. doi: 10.1371/journal.pone.0008746 (PMC2808337; doi:10.1371/journal.pone.0008746)
Supplement: Figure S2 — ATPase activity of control (vv) L. lactis membranes or of L. lactis membranes containing HMA1 or HMA6. The ATPase assay mixture contained 250 mM Tris, pH 7, 15 mM MgSO4, 15 mM ATP, 20 mM Cysteine, 100 µM TCEP, 2 µM CuSO4, and 2 µg of membrane proteins. ATPase activity was followed for 10 min at 37°C. Released inorganic phosphate was colorimetrically determined (Lanzetta et al., 1979). Data are the mean of three independent experiments and standard error to the mean are displayed. (0.27 MB DOC) [file pone.0008746.s002.doc]

HMA6

Control

**Figure S2. ATPase activity of control (vv) *L. lactis* membranes or of *L. lactis* membranes containing HMA1 or HMA6.** The ATPase assay mixture contained 250 mM Tris, pH 7, 15 mM MgSO_4_, 15 mM ATP, 20 mM Cysteine, 100 µM TCEP, 2 µM CuSO4, and 2 µg of membrane proteins. ATPase activity was followed for 10 min at 37°C. Released inorganic phosphate was colorimetrically determined (Lanzetta *et al*., 1979). Data are the mean of three independent experiments and standard error to the mean are displayed.

Lanzetta PA, Alvarez LJ, Reinach PS, Candia OA (1979) An improved assay for nanomole amounts of inorganic phosphate. Anal Biochem 100: 95-97.
