# Supplementary material for: Lactococcus lactis, an Alternative System for Functional Expression of Peripheral and Intrinsic Arabidopsis Membrane Proteins
Source: PLoS One. 2010 Jan 20;5(1):e8746. doi: 10.1371/journal.pone.0008746 (PMC2808337; doi:10.1371/journal.pone.0008746)
Supplement: Figure S3 — Analysis by SDS-PAGE of recombinant HisceQORH and Strep-tagged ceQORH proteins. A. Analysis by Coomassie blue-stained SDS-PAGE of the recombinant HisceQORH protein produced in L. lactis and E. coli. Increasing amounts of the two recombinant proteins (concentration range: 3, 6 and 9 µg) were loaded on the same gel. Note that the higher proportion of the ceQORH protein in protein extracts from L. lactis has an impact on the cleanness of the purified recombinant protein. B. Analysis by Coomassie blue-stained SDS-PAGE of the recombinant Strep-tagged ceQORH proteins (2 µg) produced in L. lactis without or with the N-ter extention (Ø att and att) encoded by the attB sites. As a control, increasing amounts of the recombinant His-tagged ceQORH protein (concentration range: 1, 2 and 4 µg) produced in L. lactis were loaded on the same gel. (0.28 MB DOC) [file pone.0008746.s003.doc]

**kDa**

118

85

49

36

26

20

**HisceQORH from *E. coli***

**HisceQORH from *L. lactis***

3 6 9 MW 3 6 9 **µg**

**A**


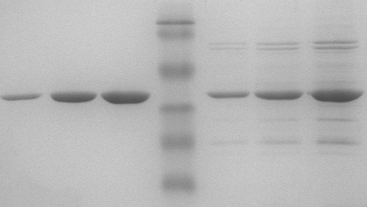


2 2 MW 1 2 4 **µg**

**ceQORH Strep from**

***L. lactis***

Ø att att

**HisceQORH from**

***L. lactis***

**B**

**kDa**

118

85

49

36

26

20


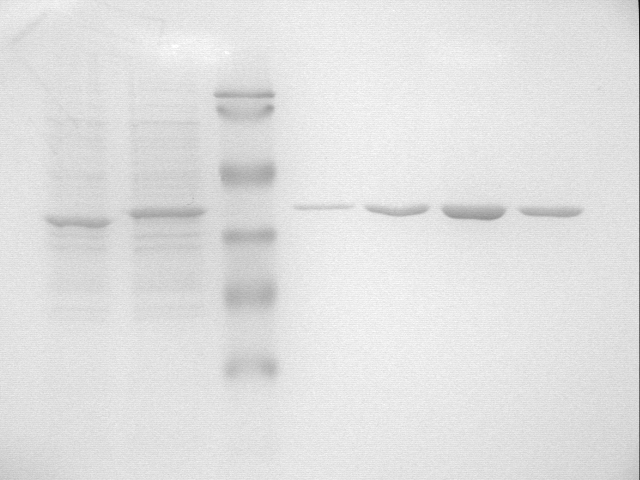


**Figure S3.** Analysis by SDS-PAGE of the recombinant HisceQORH and Strep-tagged ceQORH proteins **A.** Analysis by Coomassie blue-stained SDS-PAGE of the recombinant HisceQORH protein produced in *L. lactis* and *E. coli*. Increasing amounts of the two recombinant proteins (concentration range: 3, 6 and 9 µg) were loaded on the same gel. Note that the higher proportion of the ceQORH protein in protein extracts from *L. lactis* has an impact on the cleanness of the purified recombinant protein**.** **B.** Analysis by Coomassie blue-stained SDS-PAGE of the recombinant Strep-tagged ceQORH proteins (2 µg) produced in *L. lactis* without or with the *N*-ter extention (Ø att and att) encoded by the *att*B sites. As a control, increasing amounts of the recombinant His-tagged ceQORH protein (concentration range: 1, 2 and 4 µg) produced in *L. lactis* were loaded on the same gel.
