# Supplementary material for: Lactococcus lactis, an Alternative System for Functional Expression of Peripheral and Intrinsic Arabidopsis Membrane Proteins
Source: PLoS One. 2010 Jan 20;5(1):e8746. doi: 10.1371/journal.pone.0008746 (PMC2808337; doi:10.1371/journal.pone.0008746)
Supplement: Figure S4 — Chromatographic separation of the ceQORH protein expressed in L. lactis. The recombinant ceQORH protein was first extracted from L. lactis membranes using a salt treatment (1 M NaCl) and then further purified using an affinity chromatography. The purified recombinant ceQORH protein (1.5 mg) was then loaded onto a Superdex 200 10/300 GL column (Pharmacia Biotech) in a buffer containing 20 mM Mops pH 7.8, 300 mM NaCl and 1 mM DTT. Calibration was performed (in the same buffer) using standard proteins from GE Healthcare (the gel filtration calibration kit contains Conalbumin 75 kDa, Ovalbumin 43 kDa, Carbonic anhydrase 29 kDa and Ribonuclease A 13.7 kDa). The ceQORH protein was recovered in three fractions (12.48 ml, 14.58 ml and 15.88 ml) corresponding to apparent molecular masses of 30 kDa, 55 kDa and 148 kDa. Knowing that the expected molecular mass of the monomeric form of ceQORH is 34 kDa (Miras et al., 2002), this result suggests that the recombinant ceQORH protein produced in L. lactis might behave, in presence of salt and DTT, as a mix of momoners, dimers and tetramers. The used calibration curve is presented in B. (0.29 MB DOC) [file pone.0008746.s004.doc]

**A**

ceQORH

dimer

ceQORH

tetramer

ceQORH monomer

Void

volume

75 kDa

13.7 kDa

29 kDa

43 kDa

**B**

**Figure S4.** Chromatographic separation of the recombinant ceQORH protein produced in *L. lactis*. The recombinant ceQORH protein was first extracted from *L. lactis* membranes using a salt treatment (1 M NaCl) and then further purified using an affinity chromatography. The purified recombinant ceQORH protein (1.5 mg) was then loaded onto a Superdex 200 10/300 GL column (Pharmacia Biotech) in a buffer containing 20 mM Mops pH 7.8, 300 mM NaCl and 1 mM DTT.

Calibration was performed (in the same buffer) using standard proteins from GE Healthcare (the gel filtration calibration kit contains Conalbumin 75 kDa, Ovalbumin 43 kDa, Carbonic anhydrase 29 kDa and Ribonuclease A 13.7 kDa). The ceQORH protein was recovered in three fractions (12.48 ml, 14.58 ml and 15.88 ml) corresponding to apparent molecular masses of 30 kDa, 55 kDa and 148 kDa. Knowing that the expected molecular mass of the monomeric form of ceQORH is 34 kDa (Miras *et al*., 2002), this result suggests that the recombinant ceQORH protein produced in *L. lactis* might behave, in presence of salt and DTT, as a mix of momoners, dimers and tetramers. The used calibration curve is presented in B.

Miras S, Salvi D, Ferro M, Grunwald D, Garin J et al. (2002) Non-canonical transit peptide for import into the chloroplast. J Biol Chem 277: 47770-47778.
